# The burden of rare protein-truncating genetic variants on human lifespan

**DOI:** 10.1038/s43587-022-00182-3

**Published:** 2022-03-03

**Authors:** Jimmy Z. Liu, Chia-Yen Chen, Ellen A. Tsai, Christopher D. Whelan, David Sexton, Sally John, Heiko Runz

**Affiliations:** Translational Biology, Research & Development, Biogen Inc., Cambridge, MA USA

**Keywords:** Cancer models, Rare variants, Ageing

## Abstract

Genetic predisposition has been shown to contribute substantially to the age at which we die. Genome-wide association studies (GWASs) have linked more than 20 loci to phenotypes related to human lifespan^1^. However, little is known about how lifespan is impacted by gene loss of function. Through whole-exome sequencing of 352,338 UK Biobank participants of European ancestry, we assessed the relevance of protein-truncating variant (PTV) gene burden on individual and parental survival. We identified four exome-wide significant (*P* < 4.2 × 10^−7^) human lifespan genes, *BRCA1*, *BRCA2*, *ATM* and *TET2*. Gene and gene-set, PTV-burden, phenome-wide association studies support known roles of these genes in cancer to impact lifespan at the population level. The *TET2* PTV burden was associated with a lifespan through somatic mutation events presumably due to clonal hematopoiesis. The overlap between PTV burden and common variant-based lifespan GWASs was modest, underscoring the value of exome sequencing in well-powered biobank cohorts to complement GWASs for identifying genes underlying complex traits.

## Abstract (web summary)

This study demonstrates that the burden of protein-truncating variants identified through population-scale exome sequencing impacts how long we live. The authors report four distinct human lifespan genes with roles in cancer and clonal hematopoiesis.

## Main

Human lifespan is a heritable quantitative trait that reflects a mix of health-related outcomes, environmental exposures and chance. Twin and pedigree studies suggest that narrow-sense heritability of human lifespan ranges from 15% to 30%^2,3^. The ability to identify genetic loci associated with lifespan has been limited by the lack of mortality information in most cohorts. Instead, to approximate lifespan, GWASs have used extreme longevity or parental lifespan as outcomes. This has led to the identification of over 20 loci, several of which overlap age-related complex disease loci for Alzheimer’s disease (*APOE*), lung cancer (*CHRNA3/5*), cardiometabolic (*LPA*, *LDLR*) and immune-related disorders (*HLA*, *MAGI3*)^4–9^.

Rare PTVs are reported to have outsized effects on complex traits compared with common noncoding variants^10^ but are poorly captured on GWAS genotyping arrays. PTVs typically shorten a protein’s coding sequence by introducing premature stop codons, frameshifts or aberrant splicing that leads to partial or complete loss of its function. Individuals who carry PTVs can be considered as ‘experiments of nature’ that provide insights into gene function and allow extrapolations on efficacy and safety when a gene product is inhibited pharmacologically by drugs^11,12^.

Using whole-exome sequencing (WES) data initially from 302,331 UK Biobank participants (Online Methods), we assessed the impact of gene PTV burden on individual and parental survival using Cox’s proportional hazard models. We chose survival rather than age at death because this allowed us to account for censored data as most UK Biobank participants are still alive. For the 238,239 individuals available for analysis, 15,605 had died with their age at death recorded at the censoring dates. Fathers’ and mothers’ ages at death were reported for 178,443 and 145,281 participants, respectively. For each gene individually, as well as exome wide, we collapsed PTVs into a single score and performed burden survival analysis by testing the association between this score and six outcomes: individual lifespan, individual lifespan in males, individual lifespan in females, mother’s lifespan, father’s lifespan and mother + father combined lifespan. The most strongly associated genes were taken forward for replication in 114,099 additional exome-sequenced UK Biobank participants, of whom 7,421 have died.

**Fig. 1:**
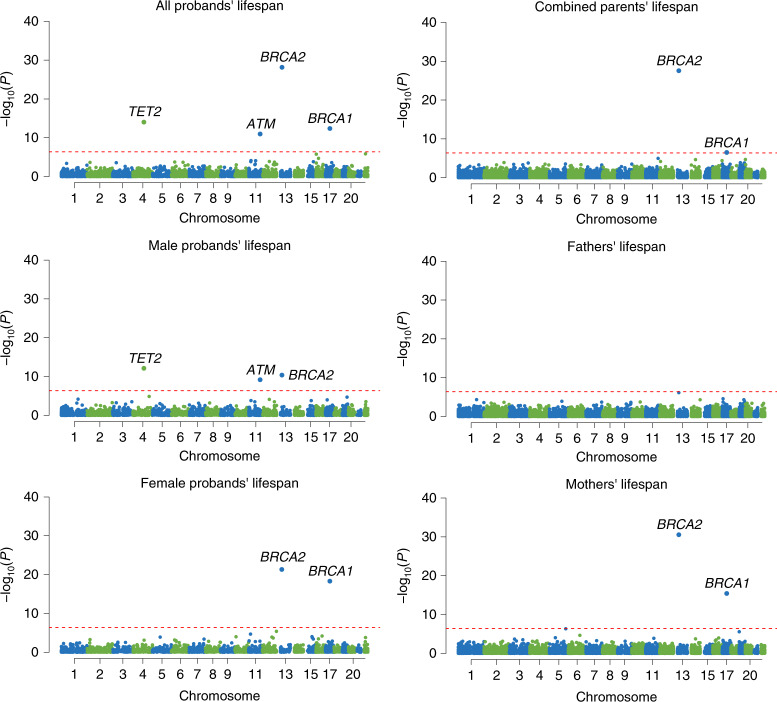
Manhattan plots of gene-based PTV-burden survival analyses in the discovery cohort of 238,239 UK Biobank participants for six survival phenotypes analyzed. Each point represents a gene. The red-dashed line indicates the exome-wide significance threshold of *P* < 4.17 × 10^−7^. Genes exceeding this threshold are labeled and colored in red. *ATM* is displayed for the two outcomes where it exceeded exome significance when results from discovery analyses were combined with the replication cohort.

**Fig. 2:**
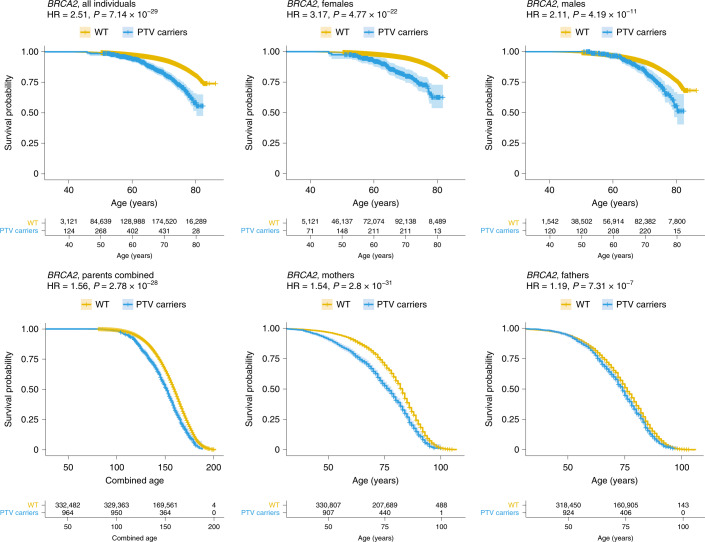
Kaplan–Meier curves for *BRCA2* PTV-burden survival analyses across the six survival phenotypes analyzed in the present study in the combined discovery and replication cohorts. Each cross represents a right censored observation. The shaded areas represent the 95% confidence interval of the survival curve. See Extended Data Fig. 1 for plots of *BRCA1*, *TET2* and *ATM*.

In the discovery cohort, we identified five genes associated with at least one survival outcome at exome-wide significance (*P* < 4.17 × 10^−7^) (Fig. 1 and Table 1). The strongest signal was observed for *BRCA2*, where PTV burden was associated with reduced lifespan in five of the six outcomes analyzed (Fig. 2): across all individuals (hazard ratio (HR) = 2.57, *P* = 3.54 × 10^−21^), in both females (HR = 3.04, *P* = 8.20 × 10^−14^) and males (HR = 2.29, *P* = 1.00 × 10^−9^) separately, and for combined parental (HR = 1.60, *P* = 2.30 × 10^−22^) and mothers’ lifespan (HR = 1.60, *P* = 4.70 × 10^−26^). *BRCA1* was associated with mothers’ lifespan (HR = 1.64, *P* = 5.35 × 10^−11^); 139 of 190 *BRCA2* PTVs and 73 of 98 *BRCA1* PTVs identified in our study had previously been reported as pathogenic or likely pathogenic in Clinvar^13^ for heritable breast and ovarian cancer syndrome or related cancers (Supplementary Table 1). Further genes exceeding exome-wide significance during discovery were *TET2* (HR = 1.76, *P* = 8.06 × 10^−10^), *PPM1D* (HR = 2.72, *P* = 1.25 × 10^−8^) and *DEDD2* (HR = 4.20, *P* = 2.28 × 10^−7^). Several other established autosomal-dominant disease genes reached nominal significance, including *LDLR* (HR = 2.28, *P* = 5.14 × 10^−7^), *ATM* (HR = 1.65, *P* = 5.26 × 10^−5^), *PALB2* (HR = 2.32, *P* = 4.06 × 10^−5^), *MLH1* (HR = 3.00, *P* = 2.09 × 10^−5^) and *PKD1* (HR = 2.58, *P* = 1.41 × 10^−5^) (Supplementary Tables 1 and 2).

**Table 1:** Survival analysis results for genes that exceeded exome-wide significance in at least one of the six survival phenotypes analyzed in the present study in either the discovery or the discovery plus replication (combined) cohort

Gene	Survival phenotype	*n* carriers^a^ (combined)	*n* PTVs^b^ (combined)	HR (discovery)	*P*^c^ (discovery)	HR (combined)	*P*^c^ (combined)
*BRCA2*	All proband	1,013	235	2.57	3.54 × 10^−21^	2.51	7.14 × 10^−29^
	Females	527	167	3.04	8.20 × 10^−14^	3.17	4.77 × 10^−22^
	Males	486	157	2.29	1.00 × 10^−9^	2.11	4.19 × 10^−11^
	Combined parents	1,005	234	1.60	2.30 × 10^−22^	1.56	2.78 × 10^−28^
	Mothers	988	232	1.60	4.70 × 10^−26^	1.54	2.80 × 10^−31^
	Fathers	975	225	1.21	1.35 × 10^−5^	1.19	7.31 × 10^−7^
*BRCA1*	All proband	411	130	2.36	8.03 × 10^−7^	2.55	4.12 × 10^−13^
	Females	194	78	3.81	5.91 × 10^−7^	4.98	4.93 × 10^−19^
	Males	217	88	1.84	7.98 × 10^−3^	1.68	5.16 × 10^−3^
	Combined parents	399	127	1.41	5.26 × 10^−5^	1.40	3.17 × 10^−7^
	Mothers	392	127	1.64	5.35 × 10^−11^	1.62	3.79 × 10^−16^
	Fathers	390	123	1.04	5.81 × 10^−1^	1.01	8.37 × 10^−1^
*TET2*	All proband	928	555	1.76	8.06 × 10^−10^	1.84	9.44 × 10^−15^
	Females	479	330	1.48	2.35 × 10^−2^	1.59	7.86 × 10^−4^
	Males	449	320	1.91	3.08 × 10^−9^	1.98	7.51 × 10^−13^
	Combined parents	917	548	0.95	2.24 × 10^−1^	0.96	3.40 × 10^−1^
	Mothers	901	541	0.95	2.83 × 10^−1^	0.97	3.32 × 10^−1^
	Fathers	886	529	0.97	4.30 × 10^−1^	1.01	7.88 × 10^−1^
*ATM*	All proband	1008	277	1.65	5.26 × 10^−5^	1.90	1.07 × 10^−11^
	Females	556	181	1.52	3.67 × 10^−2^	1.65	1.43 × 10^−3^
	Males	452	180	1.75	3.98 × 10^−4^	2.08	6.53 × 10^−10^
	Combined parents	995	273	1.17	2.20 × 10^−3^	1.14	1.44 × 10^−3^
	Mothers	979	271	1.14	6.41 × 10^−3^	1.12	5.29 × 10^−3^
	Fathers	960	266	1.09	3.73 × 10^−2^	1.11	4.66 × 10^−3^
*PPM1D*	All proband	173	63	2.72	1.25 × 10^−8^	1.95	2.48 × 10^−4^
	Females	76	38	3.35	1.36 × 10^−5^	2.40	2.51 × 10^−3^
	Males	97	50	2.41	1.08 × 10^−4^	1.73	1.91 × 10^−2^
	Combined parents	168	62	1.04	6.86 × 10^−1^	1.22	2.29 × 10^−2^
	Mothers	165	62	1.00	9.69 × 10^−1^	1.11	1.98 × 10^−1^
	Fathers	161	59	1.08	4.19 × 10^−1^	1.18	4.05 × 10^−2^
*DEDD2*	All proband	21	12	5.43	3.41 × 10^−3^	2.97	5.97 × 10^−2^
	Females	12	8	3.57	2.04 × 10^−1^	2.18	4.35 × 10^−1^
	Males	9	6	7.32	4.90 × 10^−3^	3.60	7.01 × 10^−2^
	Combined parents	21	12	2.66	1.19 × 10^−3^	1.20	4.91 × 10^−1^
	Mothers	21	12	4.20	2.28 × 10^−7^	1.36	2.15 × 10^−1^
	Fathers	20	12	1.19	5.51 × 10^−1^	0.91	7.12 × 10^−1^

^a^Number of individuals who carry at least one PTV.

^b^Number of unique PTVs.

^c^*P* value.

**Extended Data Fig. 1:**
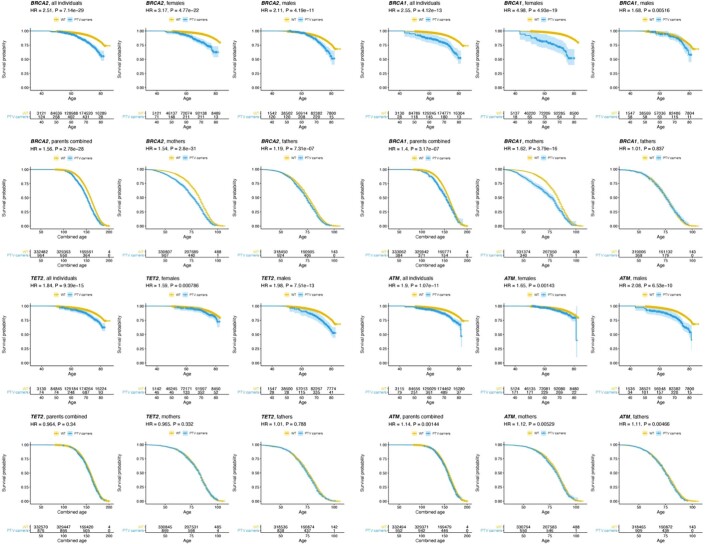
Kaplan Meier plots for *BRCA2*, *BRCA1*, *TET2* and *ATM* in the discovery + replication analysis. Kaplan Meier plots for *BRCA2*, *BRCA1*, *TET2* and *ATM* in the discovery + replication analysis. The shaded areas represent the 95% confidence interval of the survival curve.

We utilized exomes from an additional 114,099 UK Biobank participants for replication analysis of 28 genes with *P* < 0.0001 in at least one survival outcome (Supplementary Table 2). In a combined analysis in 352,338 participants, four genes, *BRCA2*, *BRCA1*, *TET2* and *ATM*, met exome-wide significance in the combined analysis, each for at least 2 of the 6 outcomes measured (Table 1 and Extended Data Fig. 1). Our analyses further supported previous findings^14^ that a higher exome-wide burden of PTVs is associated with reduced lifespan (HR = 1.0012, *P* = 2.74 × 10^−6^), and observed an even larger effect size when only considering genes with high loss-of-function (LoF) intolerance (HR = 1.07, *P* = 8.87 × 10^−17^; Supplementary Table 3).

**Extended Data Fig. 2:**
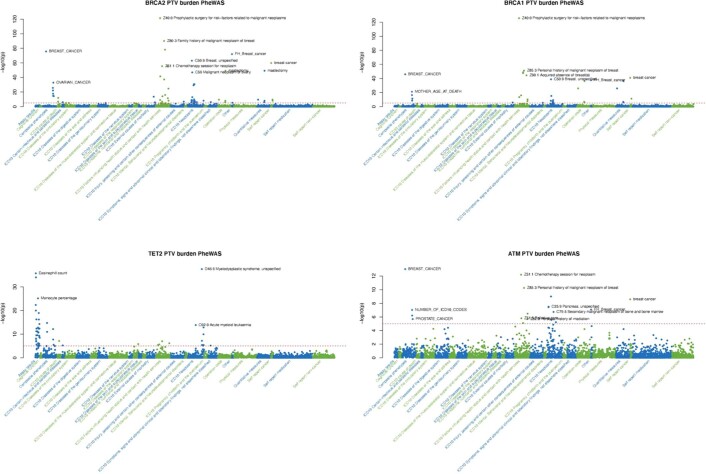
Phenome-wide association plots for *BRCA2*, *BRCA1*, *TET2* and *ATM* in all probands. Phenome-wide association plots for *BRCA2*, *BRCA1*, *TET2* and *ATM* in all probands. Each point represents a phenotype in the UK Biobank. The y-axis shows the strength of association, while the x-axis orders phenotypes by categories and then alphabetically.

To gain insights into disease endpoints and biological processes underlying the lifespan associations, we performed PTV-burden phenome-wide association studies (PheWASs) for each of the four replicated exome-wide significant genes across 4,130 semiautomatically derived UK Biobank phenotypes. Consistent with established roles in cancer^15^, *BRCA2*, *BRCA1* and *ATM* PTV burdens were associated with increased risk of breast (*BRCA2*: odds ratio (OR) = 5.85, *P* = 1.45 × 10^−71^; *BRCA1*: OR = 9.12, *P* = 7.88 × 10^−47^, ATM: OR = 2.70, *P* = 6.47 × 10^−14^) and ovarian cancer (*BRCA2*: OR = 9.33, *P* = 1.29 × 10^−33^; *BRCA1*: OR = 13.96, *P* = 3.74 × 10^−12^) in females, whereas *BRCA2* was associated with prostate cancer in males (OR = 3.47, *P* = 3.55 × 10^−15^) (Supplementary Table 4 and Extended Data Fig. 2). Next, we combined PTVs across genes annotated for similar functions and conducted gene-set burden survival analyses. Using gene-set definitions from 4,589 ConsensusPathDB pathways^16^, we identified 41 pathways associated with lifespan at a 5% Bonferroni’s threshold (*P* < 1.09 × 10^−5^). All significant pathways were linked to cancer susceptibility (Supplementary Table 5). After exclusion of *BRCA2*, *BRCA1*, *TET2* and *ATM*, 38 pathways remained nominally significant (*P* < 0.05), suggesting that further genes therein will reach gene-level significant association with lifespan when sample sizes for PTV-burden analyses increase further.

**Extended Data Fig. 3:**
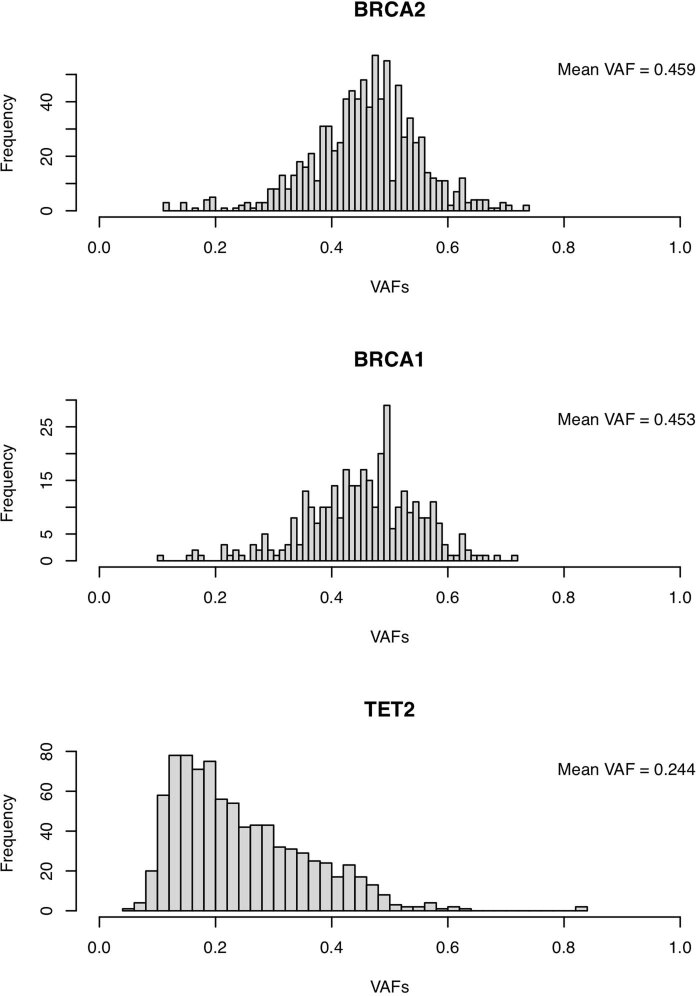
Frequency distribution of variant allele fractions of PTVs in *BRCA2*, *BRCA1* and *TET2* heterozygote carriers. Data Figure 3. Frequency distribution of variant allele fractions of PTVs in *BRCA2*, *BRCA1* and *TET2* heterozygote carriers.

**Extended Data Fig. 4:**
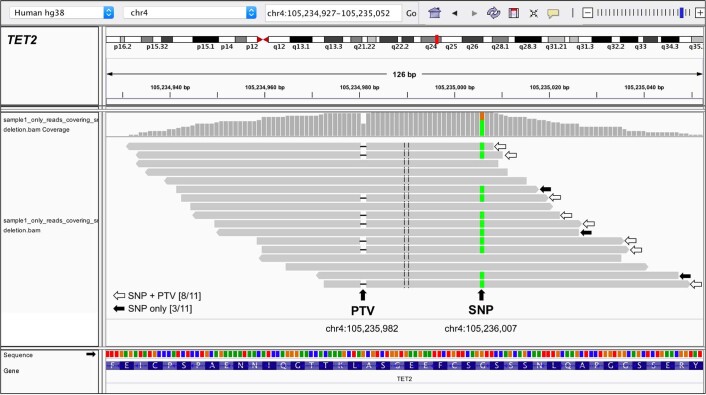
Example of a region where a common SNP (in green at chr4:105,236,007) is in close proximity to a *TET2* PTV. Example of a region where a common SNP (in green at chr4:105,236,007) is in close proximity to a *TET2* PTV. Sequencing reads spanning both variants are consistent with the PTV (the 1-bp deletion at chr4:105,235,982), being somatic in origin.

Somatic mutations in *TET2* drive clonal hematopoiesis of indeterminate potential (CHIP), the competitive expansion of a distinct bone marrow hematopoietic stem cell clone^17,18^. Our PheWASs revealed *TET2* PTV burden as associated with reduced eosinophil (β = −0.42 s.d., *P* = 2.44 × 10^−30^) and neutrophil counts (β = −0.29 s.d., *P* = 9.13 × 10^−14^) among others, along with an increased risk for myelodysplastic syndrome (OR = 16.06, *P* = 9.59 × 10^−37^), agranulocytosis (OR = 4.29, *P* = 4.78 × 10^−13^), thrombocytopenia (OR = 7.15, *P* = 4.63 × 10^−11^) and acute myeloid leukemia (OR = 12.01, *P* = 2.39 × 10^−9^) (Supplementary Table 4 and Extended Data Fig. 2). Consistent with CHIP, sequencing reads with *TET2* PTVs were highly left shifted relative to the wild-type alleles, with a mean variant allele frequency (VAF) of 0.24 across carriers (Extended Data Fig. 3). In contrast, VAFs for *BRCA2* and *BRCA1* PTVs were 0.46 and 0.45, respectively, consistent with heterozygous germline variants. A somatic origin of *TET2* PTVs was further supported through phasing with heterozygous common SNPs (Extended Data Fig. 4). As *ATM* and *PPM1D* have also been implicated in CHIP^19,20^, our results support clonal hematopoiesis as an important contributor to the genetic underpinnings of the human lifespan.

**Extended Data Fig. 5:**
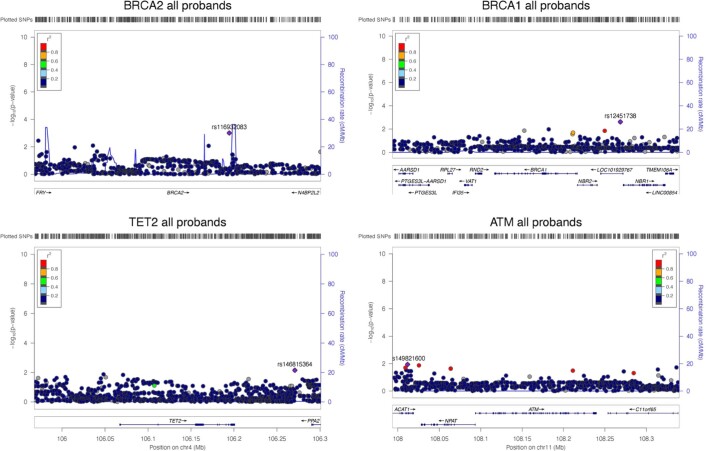
Regional association plots from survival analysis in probands at imputed and directly genotyped variants near *BRCA2*, *BRCA1*, *TET2* and *ATM*. Regional association plots from survival analysis in probands at imputed and directly genotyped variants near *BRCA2*, *BRCA1*, *TET2* and *ATM*

Notably, no common variants in *BRCA2*, *BRCA1*, *ATM* or *TET2* have been linked to lifespan through GWASs. Nor did we identify any associations between common variants and lifespan at these loci using a survival approach (Extended Data Fig. 5), although we were able to replicate 18 previously identified GWASs of parental age-at-death variants (*P* < 0.025; Supplementary Table 6)^1^. Of the 22 proposed GWAS genes, only *LDLR*, *MICB* and *SEMA6B* were nominally significant in our PTV-burden survival analyses (Supplementary Table 7).

The results of our study reflect the demographic makeup of the UK Biobank and may not fully extrapolate to other populations. The median age at censoring date in our WES sample was 67, whereas the median age at death in the UK is 82 and 85 years for males and females, respectively^21^. As such, causes of death that do not typically affect middle-aged individuals are underrepresented. Also, due to lower participation and a lack of mortality data from other ethnicities, our results are based on white European individuals and thus may not translate to all ancestries. Moreover, UK Biobank participants are healthier than the general UK population, with participants being less likely to smoke, be obese or drink^22^, which potentially dilutes our ability to capture the effects of these factors on mortality. Use of parental lifespan as a proxy phenotype reduces these ascertainment biases, although compared with directly observed phenotypes this approach requires much larger sample sizes for reaching similar statistical power^23^. Genetic associations with parental lifespan that were not detected in probands may also reflect recent advances in medical care and other environmental factors.

UK Biobank demographics cannot fully account for the limited overlap between our PTV-burden results and loci identified by previous ageing GWASs, which also included UK Biobank participants^6–8^. Instead, GWAS signals might be driven by mechanisms unrelated to protein loss of function, or act via other genes at the same locus or in *trans*. Also, at current sample sizes, PTV-based burden analyses remain underpowered to detect associations for many genes due to a lack of observations in populations that are deprived from high-impact, loss-of-function variants due to purifying selection. Nevertheless, our analyses identified robust signals for genes to impact lifespan that GWASs were yet unable to detect, such as *BRCA2*, demonstrating the potential for gene-based analyses in large sequenced biobank cohorts to complement common variant GWASs.

In conclusion, using WES data from 352,338 UK Biobank participants, we identified and replicated four genes for which loss of function is associated with human lifespan. Future efforts may expand our approach to gain of function and other rare protein-coding alleles and incorporate additional factors that impact a person’s age at death, which in most cases will be a reflection of their past health and lifestyle. Our study establishes the importance of individual genes and pathways on human lifespan at the population level and highlights intervention points that, if adequately addressed, may allow for greater wellbeing as we age.

## Methods

### UK Biobank

The UK Biobank is a prospective study of over 500,000 participants aged 40–69 years recruited from 2006 to 2010 in the UK^24^. Participant data include health records, medication history and self-report survey information along with imputed genome-wide genotypes^25^. Further details are available at https://biobank.ndph.ox.ac.uk/showcase. Analyses in the present study were conducted under UK Biobank approved project no. 26041. The UK Biobank has approval from the North West Multi-centre Research Ethics Committee, which covers the UK. It also sought the approval in England and Wales from the Patient Information Advisory Group (PIAG) for gaining access to information that would allow it to invite people to participate. PIAG has since been replaced by the National Information Governance Board for Health and Social Care. In Scotland, UK Biobank has approval from the Community Health Index Advisory Group. UK Biobank possesses a Human Tissue Authority (HTA) license, so a separate HTA license is not required by researchers who receive samples from the resource, so long as residual samples have been destroyed or returned at the end of the research project, and applicants do not transfer the samples to third party premises without the specific approval of UK Biobank. UK Biobank has sought generic Research Tissue Bank approval, which should cover the large majority of research using the resource. This approach is recommended by the National Research Ethics Service and UK Biobank governing Research Ethics Committee, which approved the application in 2010. Researchers should check the UK Biobank Access Procedures for more detail.

WES data for UK Biobank participants were generated at the Regeneron Genetics Center (RGC) as part of a collaboration of AbbVie, Alnylam Pharmaceuticals, AstraZeneca, Biogen, Bristol-Myers Squibb, Pfizer, Regeneron and Takeda with the UK Biobank^26^. WES data were processed using the pipeline described in Szustakowski et al.^26^. RGC generated a quality control (QC)-passing ‘Goldilocks’ set of 23,482,637 genetic variants from a total of 302,331 sequenced UK Biobank participants. For replication, we used WES data generated from the same RGC pipeline in an additional 152,486 UK Biobank participants.

### Survival phenotypes

UK Biobank participants’ ages at death (UKB Data-Field 40007) were automatically linked through NHS Digital (for England and Wales) and Information and Statistics Division (for Scotland), and were current as of June 2020, which was used as the censoring date^27^. For individuals without a death record, we assumed they were alive on the censoring date, and calculated their current age to be June 2020 minus their year and month of birth.

During the initial UK Biobank (UKB) assessment between 2006 and 2010, all participants were asked to record the ages of their father/mother if alive (UKB Data-Fields 1797, 2946, 1835 and 1845), or their parents’ respective age at death (Data-Fields 1807 and 3526). Participants were also asked whether they were adopted as a child (Data-Field 1767). Repeat assessment (2012 onward) data were available for 56,378 participants. We extracted the parental ages/ages at death from the most recently provided assessment visit of each participant.

### Gene and PTV annotation

Variants identified through WES were annotated with VEP v.96 (ref. ^28^) and the LOFTEE^12^ plugin. LOFTEE applies a range of filters on stop-gained, splice-site disrupting and frameshift variants to exclude putative PTVs due to variant annotation and sequencing mapping errors that are unlikely to substantially disrupt gene function. For instance, stop-gained and frameshift variants that are within 50 kb of the end of the transcript will be flagged as ‘low confidence’. We extracted variants predicted as PTVs, flagged as ‘high confidence’ by LOFTEE and with minor allele frequency (MAF) <1% in UK Biobank participants of white British ancestry (below) for each canonical transcript (as defined in Ensembl). We identified 572,780 high-confidence predicted rare PTVs (MAF < 1%), including 386,785 singletons, in the canonical transcripts of 19,094 genes. Each individual carried on average 19 rare PTVs, which is consistent with previous estimates^12,26,29^. Genes with high LoF intolerance were defined as those with probability of LoF intolerance >0.9, as calculated in the gnomAD cohort^12^.

### Survival analysis

We restricted our analyses to the 88.1% of UK Biobank participants with ‘Caucasian’ genetic ethnic grouping based on principal component analysis (PCA) in Bycroft et al.^25^ (UKB Data-Field 22006) and those with self-reported ‘white British’ ethnic background (UKB Data-Field 21000). To account for relatedness, we excluded from our analyses one member (at random) from each pair of relatives who are second-degree relatives or lower. As analyses in the Asian or Asian British (1.96%), black or black British (1.6%) or Chinese (0.31%) subcohorts of UK Biobank, for which few mortality data were available, were insufficiently powered (not shown), we also excluded non-white British ancestry participants based on PCA of the public genotype data^25^.

A total of 238,239 individuals was available for discovery analyses, of whom 9,405 had died with their age at death recorded at the censoring date. For the parental survival analysis, we adopted a similar approach to previous studies using the UK Biobank parental information^8^ and further excluded adopted individuals (*n* = 3,090), individuals who did not know/did not answer the parental age/age-at-death questions (7,383 fathers, 3517 mothers), or when a parent had died before the age of 40 (4,174 fathers, 2,571 mothers). Fathers’ and mothers’ ages at death were reported for 178,443 and 145,281 participants, respectively, whereas 56,706 and 89,686 participants reported the age of their fathers and mothers, respectively, as being alive at the time of recruitment or follow-ups. The survival analysis in fathers included 235,149 individuals and 178,443 events and that in mothers included 234,967 individuals and 145,281 events. We created a combined father and mother age by summing the reported ages of the parents and defining events as whether both parents had died (*n* = 225,701, *n* events = 128,045).

**Extended Data Fig. 6:**
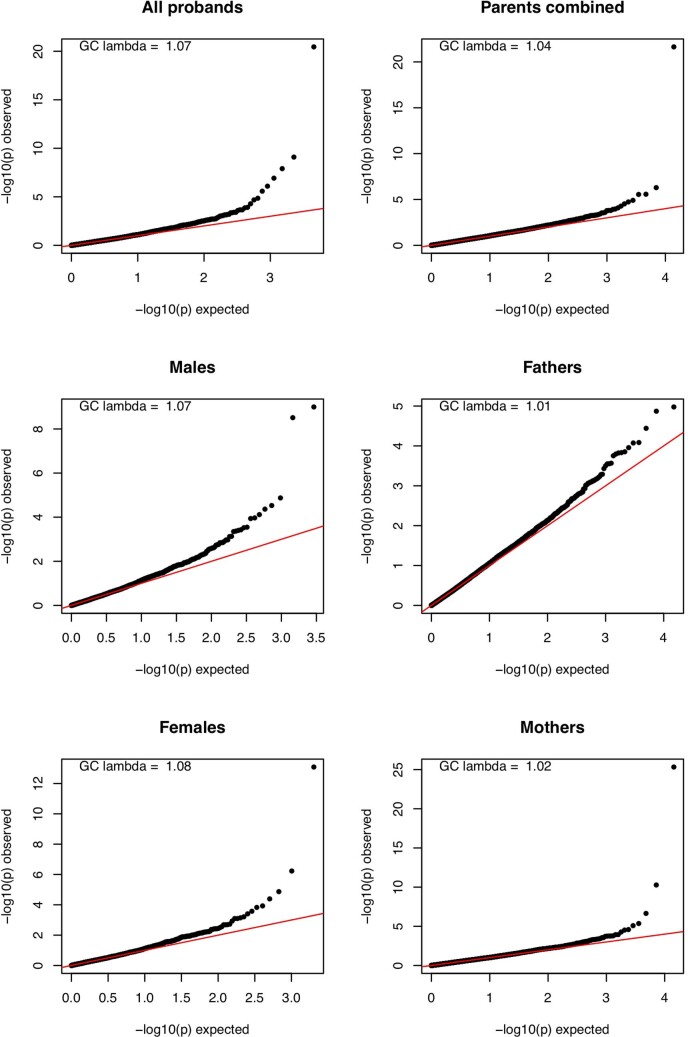
QQ-plots and genome control (GC) lambdas from the PTV burden analyses across six survival outcomes. QQ-plots and genome control (GC) lambdas from the PTV burden analyses across six survival outcomes

**Extended Data Fig. 7:**
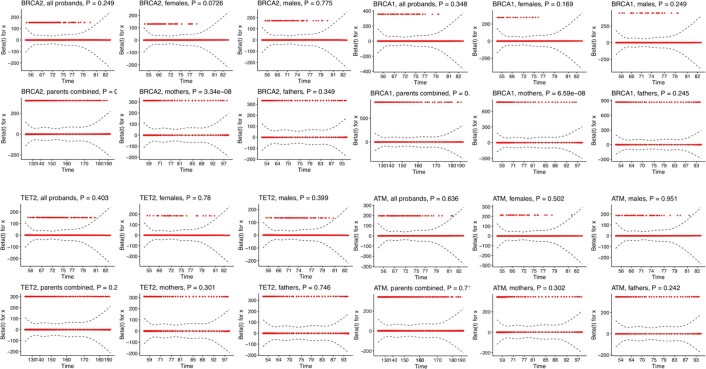
Scaled Schoenfeld residuals of the PTV status (*x*) vs time plots for *BRCA2*, *BRCA1*, *TET2* and *ATM* burden in each of the six survival outcomes tested. Scaled Schoenfeld residuals of the PTV status (*x*) vs time plots for *BRCA2*, *BRCA1*, *TET2* and *ATM* burden in each of the six survival outcomes tested. Proportional hazards assumption test p-values for the PTV variable are shown above each plot.

For each gene, we applied Cox’s proportional hazards model (right censored) to test for an association between survival and PTV burden: $h\left( {x_i} \right) = h_0\left( {x_i} \right){\mathrm{exp}}\left( {\beta g_i + \gamma _iZ_i} \right)$, where for each individual *i*, *x* is the age, *h*_0_ is the baseline hazard, *g* is the genotype with coefficient *β* and *Z* is a matrix of covariates with coefficient *γ*. For each gene, *g*_*i*_ = 1 if individual *i* carries at least one PTV, otherwise *g*_*i*_ = 0. We included baseline age, sex and 10 principal components (PCs) as covariates in all the survival phenotypes analyzed, except for the proband sex-specific analyses, where the sex covariate was dropped. For the analysis including PTVs across all genes, *g*_*i*_ denoted the total number of PTVs carried by individual *i*. For single variant tests, *g*_*i*_ denotes the number of minor alleles carried by individual *i*. All Cox’s regressions were performed in R with the ‘survival’ package^30^. Six survival phenotypes were analyzed: proband age, male proband age, female proband age, combined parental age, father’s age and mother’s age. Approximate exome-wide significance was defined as *P* < 0.05 divided by 20,000 genes divided by 6 outcomes = 4.17 × 10^−7^. As Cox’s model may produce biased type I error estimates when the number of observed events/predictors are low^31^, we excluded genes with fewer than 10 PTVs among noncensored individuals. QQ plots were manually inspected and genomic inflation factor calculated by dividing the median χ^2^ statistics for each outcome by 0.456 (Extended Data Fig. 6). Deviations from the proportional hazards assumption were tested by visual inspection of Schoenfeld residuals and testing for a nonzero slope in a generalized linear model of Schoenfeld residuals with time (Extended Data Fig. 7).

For replication, we used WES data from an additional 114,099 UK Biobank participants and performed PTV annotation and sample exclusions using the same criteria as in the discovery phase, resulting in 352,338 individuals available for survival analysis. We applied the same survival models described previously at genes that were nominally significant in the discovery phase (*P* < 1 × 10^−4^) and those previously implicated in GWASs^1^.

To compare our survival approach against that of using uncensored ages at death, we also performed PTV-burden association tests, treating age at death or parental ages at death as quantitative phenotypes at *BRCA2*, *BRCA1*, *TET2* and *ATM* in the combined discovery and replication set, using linear regression with covariates age, sex and the first 10 PCs (Supplementary Table 7). Only *BRCA2* (*β* = −5.13 years, *P* = 2.86 × 10^−27^ in mothers’ lifespan) and *BRCA1* (*β* = −7.22 years, *P* = 6.87 × 10^−22^ in mothers’ lifespan) were exome-wide significant in any of the survival outcomes measured (Supplementary Table 8).

### PheWAS analysis

For genes that exceeded exome-wide significance in Cox’s analysis, we performed a PTV-burden PheWAS across 4,130 semiautomatically derived UK Biobank phenotypes. Binary phenotypes included *International Classification of Disease*, 10th edn (ICD-10)^32^ codes (each primary ICD-10, secondary ICD-10 and cause of death ICD-10 code were combined into a single ICD-10 phenotype), self-reported health outcomes, medication usage, surgery/operation codes and family history (fathers’ illnesses, mothers’ illnesses and siblings’ illnesses were combined into a single phenotype for each of the 12 family history illnesses ascertained in UK Biobank questionnaires). Additional disease endpoints (for example, breast cancer, ovarian cancer, type 2 diabetes) were derived manually by combining the ICD codes, self-report, medication, operation codes and other relevant UK Biobank fields. Quantitative phenotypes include 31 blood count phenotypes (for example, lymphocyte count), 30 blood biochemistry phenotypes (for example, cholesterol), 47 infectious disease antigen assays (for example, L1 antigen for human papilloma virus), and >400 physical (for example, hand-grip strength) and cognitive (for example, numeric memory) measurements.

We excluded binary phenotypes with <100 cases among the 352,338 post-QC set of UK Biobank participants. PTV-burden testing for binary phenotypes was performed using logistic regression in all individuals as well as males and females separately. For gene–phenotype pairs where the PTV-burden *P* value <0.05, we repeated the analysis using the Firth method to account for situations where the logistic regression outputs may be biased due to separation^33^. For quantitative phenotypes, we excluded phenotypes with <500 observations. For each phenotype, outlying individuals (defined as having >5 s.d. from the mean) were excluded. Burden testing was performed using linear regression on both the raw and the inverse rank normal transformed quantitative phenotypes in all individuals, as well as males and females separately. In both the logistic and linear models, we included covariates age, sex and 10 PCs. Sex was excluded as a covariate for the sex-specific analyses. We defined a 5% Bonferroni’s corrected, phenome-wide significance threshold of *P* < 1.2 × 10^−5^ (= 0.05/4,130).

### Gene-set PTV survival analysis

We performed gene-set PTV survival analysis by first grouping genes into pathways as defined by ConsensusPathDB-human release 30 (ref. ^16^), which integrates >4,589 pathways from 32 databases including KEGG (Kyoto Encyclopedia of Genes and Genomes), BioCarta and WikiPathways. For each pathway, we collapsed PTVs from all genes that are annotated as being members of a respective pathway into a single score and tested this score for association with survival using the same Cox’s model approach as described above for individual genes.

### Confirmation of somatic PTVs at *TET2*

To investigate the potential for clonal hematopoiesis driving the significant associations between *TET2* PTVs and survival, we first compared the distribution of the alternative VAFs of the PTVs in individuals who are heterozygous for PTVs at *TET2*, *BRCA2* or *BRCA1*. The VAF was calculated for each variant per individual, and is defined as the total number of reads supporting the alternative allele divided by the total number of reads supporting either the alternative or the reference allele. Multi-allelic sites were split, and we only considered the alleles predicted to be high-confidence loss of function. As it is known that germline *BRCA2* and *BRCA1* PTVs drive associations with cancer, we would expect most VAFs in these genes to be close to 50% in heterozygotes, whereas, for somatic variants, their VAFs would all be <50%.

To further confirm that PTVs in *TET2* were somatic, we systematically searched in the CRAM files of all 665 *TET2* PTV carriers for aligned reads and read pairs that spanned both a PTV and a nearby common germline SNP (defined here as an SNP with MAF > 1%). In all, five such PTV–SNP pairs were identified in four individuals. For each of the five PTV–SNP pairs, we counted the number of times the following PTV–SNP reference/alternative allele combinations was observed on the same read (or read pair): (1) PTV-ref with SNP-ref, (2) PTV-ref with SNP-alt, (3) PTV-alt with SNP-ref and (4) PTV-alt with SNP-alt; under the assumption that the PTV is somatic, we would expect to observe reads following patterns (1) and (2), which reflect cells that do not contain the somatic mutation, and only one of either (3) or (4), reflecting cells that carry the mutation that occurred once either on the SNP-ref or the SNP-alt haplotype. If, for the same individual, (3) is found with (1), or (4) is found with (2), then the PTV can be assumed to be somatic.

### Reporting Summary

Further information on research design is available in the Nature Research Reporting Summary linked to this article.

## Supplementary information

Reporting Summary.

Supplementary Table 1. Supplementary Tables 1–8.

## Data Availability

Full summary statistics from the present study are available under the following link: https://github.com/jimmyzliu/lifespan_paper. Summary and individual-level WESS data from UK Biobank participants have been deposited with UK Biobank and are freely available to approved researchers via the UK Biobank Research Analysis Platform (https://www.ukbiobank.ac.uk/enable-your-research/research-analysis-platform). Additional information about registration for access to the data is available at http://www.ukbiobank.ac.uk/register-apply. Data for the present study were obtained under Resource application no. 26041.

## Code availability

Codes used for analyses in the present study are available under the following link: https://github.com/jimmyzliu/lifespan_paper.
