# Supplementary material for: The burden of rare protein-truncating genetic variants on human lifespan
Source: Nat Aging. 2022 Mar 3;2(4):289–94. doi: 10.1038/s43587-022-00182-3 (PMC10154195; doi:10.1038/s43587-022-00182-3)
Supplement: Supplementary file 1 — Reporting Summary. [file 43587_2022_182_MOESM1_ESM.pdf]

nature research

Corresponding author(s): Jimmy Liu, Heiko Runz

Last updated by author(s): Dec 16, 2021

Reporting Summary

Nature Research wishes to improve the reproducibility of the work that we publish. This form provides structure for consistency and transparency in reporting. For further information on Nature Research policies, see Authors & Referees and the Editorial Policy Checklist.

Statistics

For all statistical analyses, confirm that the following items are present in the figure legend, table legend, main text, or Methods section.

n/a	Confirmed	
☐	☒	The exact sample size (n) for each experimental group/condition, given as a discrete number and unit of measurement
☐	☒	A statement on whether measurements were taken from distinct samples or whether the same sample was measured repeatedly
☐	☒	The statistical test(s) used AND whether they are one- or two-sided<br>*Only common tests should be described solely by name; describe more complex techniques in the Methods section.*
☐	☒	A description of all covariates tested
☐	☒	A description of any assumptions or corrections, such as tests of normality and adjustment for multiple comparisons
☐	☒	A full description of the statistical parameters including central tendency (e.g. means) or other basic estimates (e.g. regression coefficient) AND variation (e.g. standard deviation) or associated estimates of uncertainty (e.g. confidence intervals)
☐	☒	For null hypothesis testing, the test statistic (e.g. F, t, r) with confidence intervals, effect sizes, degrees of freedom and P value noted<br>*Give P values as exact values whenever suitable.*
☒	☐	For Bayesian analysis, information on the choice of priors and Markov chain Monte Carlo settings
☒	☐	For hierarchical and complex designs, identification of the appropriate level for tests and full reporting of outcomes
☐	☒	Estimates of effect sizes (e.g. Cohen's d, Pearson's r), indicating how they were calculated

Our web collection on statistics for biologists contains articles on many of the points above.

Software and code

Policy information about availability of computer code

Data collection	No specific software were employed for data collection.
Data analysis	Details of specific software and references, including genetic measurements and QC, can be found within text in the relevant Methods and Supplementary Information sections. Codes used for analyses in this study are available under the following link: https://github.com/jimmyzliu/lifespan_paper.

For manuscripts utilizing custom algorithms or software that are central to the research but not yet described in published literature, software must be made available to editors/reviewers. We strongly encourage code deposition in a community repository (e.g. GitHub). See the Nature Research guidelines for submitting code & software for further information.

Data

Policy information about availability of data

All manuscripts must include a data availability statement. This statement should provide the following information, where applicable:

- Accession codes, unique identifiers, or web links for publicly available datasets
- A list of figures that have associated raw data
- A description of any restrictions on data availability

Full summary statistics from this study are available under the following link: https://github.com/jimmyzliu/lifespan_paper. Summary and individual-level whole exome sequencing data from UKB participants have been deposited with UKB and are freely available to approved researchers via The UK Biobank Research Analysis Platform (https://www.ukbiobank.ac.uk/enable-your-research/research-analysis-platform). Additional information about registration for access to the data is available at http://www.ukbiobank.ac.uk/register-apply/. Data for this study were obtained under Resource Application Number 26041.

Field-specific reporting

Please select the one below that is the best fit for your research. If you are not sure, read the appropriate sections before making your selection.

☒ Life sciences ☐ Behavioural & social sciences ☐ Ecological, evolutionary & environmental sciences

For a reference copy of the document with all sections, see nature.com/documents/nr-reporting-summary-flat.pdf

Life sciences study design

All studies must disclose on these points even when the disclosure is negative.

Sample size	Sample size was chosen based on the availability of WES data from UK Biobank. At the time of analysis, this was ~300,000 participants
Data exclusions	We restricted our analyses to the 88.1% of UK Biobank participants with "Caucasian" genetic ethnic grouping based on PCA analysis in Bycroft et al, 2018 (UKB Data-Field 22006) and those with self-reported "white-British" ethnic background (UKB Data-Field 21000). To account for relatedness, we excluded from our analyses one member (at random) from each pair of ≤2nd degree relatives. Since analyses in the Asian or Asian British (1.96%), Black or Black British (1.6%), or Chinese (0.31%) subcohorts of UK Biobank, for which little mortality data was available, were insufficiently powered (not shown), we also excluded non-white British ancestry participants based on principal components analysis of the public genotype data. Further data exclusions as part of qc are described in Methods.
Replication	Replication was not attempted for top findings since exome-sequencing data of sufficient sample sizes (i.e. similar in magnitude to UK Biobank) and with the required phenotypes are not available at this time.
Randomization	No experimental vs control group per se.
Blinding	No experimental vs control group per se. All data are anonymised and analysts were blind to sample status.

Reporting for specific materials, systems and methods

We require information from authors about some types of materials, experimental systems and methods used in many studies. Here, indicate whether each material, system or method listed is relevant to your study. If you are not sure if a list item applies to your research, read the appropriate section before selecting a response.

Materials & experimental systems

n/a	Involved in the study	
☒	☐	Antibodies
☒	☐	Eukaryotic cell lines
☒	☐	Palaeontology
☒	☐	Animals and other organisms
☐	☒	Human research participants
☒	☐	Clinical data

Methods

n/a	Involved in the study	
☒	☐	ChIP-seq
☒	☐	Flow cytometry
☒	☐	MRI-based neuroimaging

Human research participants

Policy information about studies involving human research participants

Population characteristics	Details of the UK Biobank cohort are described in Bycroft et al., 2018 and Szustakowski et al., 2021 (refs. 25 and 26)
Recruitment	Details on the UK Biobank cohort recruitment are described in Bycroft et al., 2018 and at https://www.ukbiobank.ac.uk
Ethics oversight	Analyses in this study were conducted under UK Biobank Approved Project number 26041. UK Biobank has approval from the North West Multi-centre Research Ethics Committee (MREC), which covers the UK. It also sought the approval in England and Wales from the Patient Information Advisory Group (PIAG) for gaining access to information that would allow it to invite people to participate. PIAG has since been replaced by the National Information Governance Board for Health & Social Care (NIGB). In Scotland, UK Biobank has approval from the Community Health Index Advisory Group (CHIAG). UK Biobank possesses a Human Tissue Authority (HTA) licence, so a separate HTA licence is not required by researchers who receive samples from the resource, so long as residual samples are destroyed or returned at the end of the research project, and applicants do not transfer the samples to third party premises without the specific approval of UK Biobank. UK Biobank has sought generic Research Tissue Bank (RTB) approval, which should cover the large majority of research using the resource. This approach is recommended by the National Research Ethics Service and UK Biobank governing Research Ethics Committee (REC), which approved the application in 2010. Researchers should check the UK Biobank Access Procedures for more detail.

Note that full information on the approval of the study protocol must also be provided in the manuscript.
